# Comprehensive Assessment of *TERT* mRNA Expression across a Large Cohort of Benign and Malignant Thyroid Tumours

**DOI:** 10.3390/cancers12071846

**Published:** 2020-07-09

**Authors:** Ana Pestana, Rui Batista, Ricardo Celestino, Sule Canberk, Manuel Sobrinho-Simões, Paula Soares

**Affiliations:** 1 Institute of Molecular Pathology and Immunology of the University of Porto (Ipatimup), 4200-135 Porto, Portugal; apestana@ipatimup.pt (A.P.); rbatista@ipatimup.pt (R.B.); ricardo.celestino@ibmc.up.pt (R.C.); scanberk@ipatimup.pt (S.C.); ssimoes@ipatimup.pt (M.S.-S.); 2 i3S-Instituto de Investigação e Inovação em Saúde, Universidade do Porto, 4200-135 Porto, Portugal; 3 Medical Faculty of University of Porto (FMUP), 4200-139 Porto, Portugal; 4 School of Allied Health Technologies, Polytechnic of Porto, 4200-072 Porto, Portugal; 5 Abel Salazar Biomedical Sciences Institute (ICBAS), University of Porto, 4050-313 Porto, Portugal; 6 Department of Pathology, Centro Hospitalar São João, 4200-139 Porto, Portugal; 7 Department of Pathology, Medical Faculty of the University of Porto, 4200-139 Porto, Portugal

**Keywords:** thyroid tumours, *TERT* promoter mutations, *TERT* expression, immortalization, telomerase

## Abstract

The presence of *TERT* promoter (*TERT*p) mutations in thyroid cancer have been associated with worse prognosis features, whereas the extent and meaning of the expression and activation of *TERT* in thyroid tumours is still largely unknown. We analysed frozen samples from a series of benign and malignant thyroid tumours, displaying non-aggressive features and low mutational burden in order to evaluate the presence of *TERT*p mutations and *TERT* mRNA expression in these settings. In this series, *TERT*p mutations were found in 2%, only in malignant cases, in larger cancers, and from older patients. *TERT* mRNA expression was detected in both benign and malignant tumours, with increased frequencies in the malignant tumours with aggressive histotypes, larger tumours, and from older patients. In benign tumours, *TERT* mRNA expression was found in 17% of the follicular thyroid adenoma (FTA) with increased levels of expression in smaller tumours and associated with the presence of thyroiditis. *TERT*p mutations and *TERT* mRNA expression are correlated with worse prognosis features in malignant thyroid tumours, whereas *TERT* mRNA expression in the benign tumours is associated with the presence of thyroiditis.

## 1. Introduction

Achieving unlimited proliferative potential, cell immortalization, has been considered one of the cancer hallmarks [1]. Telomerase re-expression has been detected in 80 to 90% of all human cancers [2,3] suggesting that this is a favoured mechanism in cancer cells to circumvent the proliferative barrier [1]. Telomerase is composed of several subunits, however, it has been shown that two of them are essential for telomerase catalytic activity, the telomerase reverse transcriptase (*TERT*), the catalytic subunit, and a RNA component (*TERC*) that provides a template for telomere elongation [4,5,6]. *TERC* is widely expressed in several tissues, even in telomerase negative, however, *TERT* expression is highly regulated, and a positive correlation between *TERT* mRNA expression and telomerase activity has been reported, suggesting that *TERT* is the main regulator of telomerase [7,8,9].

*TERT* promoter (*TERT*p) mutations have been identified as one of the mechanisms responsible for telomerase re-expression in cancer, being described for the first time in melanoma [10,11]. The *TERT*p mutations c.1–124 (C228T) and c.1–146 (C250T) residues (C > T or G > A in the reverse strand) [10,11], create an 11-base nucleotide stretch 5’-CCCCTTCCGGGG-3’ which contains a consensus binding site, GGAA (in reverse complement), for E-Twenty Six (ETS) family of transcription factors, providing a basis to the biological relevance of these mutations [10,11].

Telomerase re-activation and *TERT* genetic alterations, such as the *TERT*p mutations have been reported in thyroid tumours. Thyroid tumours can be classified in several histotypes and variants. More than 95% of all thyroid tumours are follicular cell derived tumours (FCDT) [12]; the most common benign tumour is the follicular thyroid adenoma (FTA), whereas malignant tumours comprise four major histotypes: the papillary thyroid carcinoma (PTC); the follicular thyroid carcinoma (FTC); the poorly differentiated thyroid carcinoma (PDTC); and the anaplastic thyroid carcinoma (ATC) [13].

Telomerase re-activation has been reported in 48% of papillary thyroid carcinoma, 71% of follicular thyroid carcinoma, and in 78% of poorly differentiated thyroid carcinoma and anaplastic thyroid carcinoma [14,15,16,17,18]. The *TERT*p mutations were mostly detected in FCDTC, such as well differentiated thyroid carcinoma (WDTC, which includes the PTC and FTC), and less differentiated thyroid carcinoma, which includes the PDTC and ATC. The presence of these mutations in malignant tumours has been associated with worse prognostic features, such as age, tumour size, and tumour stage, as well as with distant metastasis, worse response to treatment and short survival [19,20,21,22,23,24,25,26]. The *TERT*p mutations have been rarely reported in benign lesions such as multinodular goiter, FTA or thyroiditis.

Recently, *TERT*p mutations, have been reported in thyroid benign tumours [27,28,29]. Wang et al. reported the presence of *TERT*p mutations in 2% of FTA and 17% of atypical FTAs, these tumours being positive for *TERT* mRNA and telomerase activity, whereas the FTAs negative for *TERT*p mutations were negative for both features [29]. The authors suggested that the presence of these mutations occurred as an early event in thyroid tumours that still did not develop malignant features [29]. Other works have also analysed the role of *TERT*p mutations as a predictor of the disease, where they report a frequency of 39% in atypical FTAs, also designated ‘follicular tumour of uncertain malignant potential’ (FT-UMPs) [30]. In accordance, Hysek et al. reported that *TERT*p mutated FT-UMPs harboured malignant potential and exhibited similar recurrences rates as *TERT*p mutated minimally invasive FCTs [31]. Our group reported the presence of these mutations in a radiation context, in which *TERT*p mutations were detected in 12% of carcinomas and in 21% of adenomas; the mutational profile is different in the latter, being the most frequent alterations the c.1−146C > T and the c.1−124/−125CC > TT tandem mutation. In the same work, we did not detect *TERT*p mutations in sporadic adenoma non-exposed to radiation [27]. It has been demonstrated that the c.1−146C > T confers a lower telomerase transcriptional activity in comparison to the c.1−124C > T mutation [32]. In this setting the aforementioned mutations do not appear to drive carcinogenesis, since *TERT*p mutations are equally prevalent in the carcinomas arising in irradiated and in the sporadic setting [27].

In order to continue disclosing and understanding *TERT*p mutations and *TERT* mRNA expression role in thyroid tumours, a series of frozen thyroid tumours was evaluated. We verified that *TERT*p mutations are absent in sporadic benign tumours and present at low prevalence in sporadic malignant thyroid tumours. *TERT* mRNA expression was found both in benign and malignant tumours, the frequency being higher in the latter regardless of the *TERT*p mutational status. In malignant tumours, *TERT* mRNA increased expression was associated with older age and larger tumours. However, in benign tumours, *TERT* mRNA expression can be misinterpreted by the presence of lymphocytic infiltration and thyroiditis.

## 2. Results

### 2.1. TERTp Mutations Are Only Found in Malignant Thyroid Tumours

The series was composed of a total of 244 samples from 202 patients encompassing benign lesions, malignant lesions, and tumour adjacent thyroid samples. The series was composed of 11 samples of chronic lymphocytic thyroiditis (CLT), 87 samples of FTA (with available adjacent tissue in 7 cases), 13 FTC cases (with available adjacent tissue in 8 cases), 85 cases of PTC (with available adjacent tissue in 23 cases) and 5 cases of PDTC (with available adjacent tissue in 2 cases). We also analysed metastatic tissue (*n* = 3), 2 samples of lymph node metastasis one paired with a primary tumour and the other paired with a distant metastasis. The 85 PTC cases included classical variant of PTC (*n* = 48; CV-PTC), follicular variant of PTC (*n* = 21; 11 encapsulated and 10 non-encapsulated; FV-PTC), aggressive variants of PTC (*n* = 11; 3 cases of tall cell PTC, 6 cases of diffuse sclerosing PTC, and 2 cases of trabecular/solid PTC) and oncocytic variant of PTC (*n* = 5; OV-PTC). Table 1 represents the histological, clinico-pathological, and molecular parameters considered.

The *BRAF* mutations were the most frequent genetic alteration detected in the cases (22.2%), only in malignant tumours, with 77% of the mutations found in CV-PTC (20 cases), 12% in aggressive variants of PTC (1 tall cell PTC; 1 diffuse sclerosing PTC and 1 trabecular/solid PTC) and 12% in FV-PTC (3 cases). *NRAS* mutations were detected in 8.9% of the cases, being present in benign and malignant tumours, with a higher frequency in the FTA (35%—6 cases), 18% in CV-PTC (3 cases), 12% in FTC, FV-PTC and OV-PTC (2 cases each), and 6% in PDTC (1 case) and in the single distant metastasis analysed. *TERT*p mutations, were only detected in malignant cases, occurring in 2.1% of all the series, with the highest frequency of 50% in OV-PTC (2 cases), 25% in PDTC (1 case) and in the distant metastasis. *RET/PTC* rearrangements were present in 4.6% of all series, being 67% in CV-PTC (6 cases), 22% in aggressive variants of PTC (1 diffuse sclerosing PTC and 1 solid trabecular), and 11% found in FTC (1 case). Finally, the *PAX8/PPARγ* rearrangements was the least frequent alteration, with 1.6% in all the cases, 67% being detected in FTA (2 cases) and 33% in FTC (1 case). Of note, the FTC positive for the *PAX8/PPARγ* rearrangement was also positive for *RET/PTC* rearrangement.

In PTCs, *BRAF* (V600E) mutation was associated with the presence of minimal extrathyroidal extension (miETE; 59.1% of the *BRAF* mutated tumours have miETE vs. 26.1% of the *BRAF* wild type tumours; *p* = 0.004) and with the presence of lymphocytic infiltrate (66.7% of the *BRAF* mutated tumours have lymphocytic infiltration vs. 35.7% of the wild type tumours¸ *p* = 0.012).

*TERT*p mutations were present in 3 primary tumours (in 2 OV-PTCs and in 1 PDTC), all cases with the c.1–124C > T mutation, and in a distant metastasis (bone). From the same patient with the distant metastasis also one lymph node metastasis was analysed, and both were *TERT*p (c.1−146C > T) and *NRAS* (Q61R) mutated.

The three primary tumours and the distant metastasis with *TERT*p mutation (Table 2) belonged to older patients (three out four patients were older than 55 years, *p* = 0.042; mean age of 60 years, *p* = 0.039). Moreover, the presence of *TERT*p mutations occurred in lesions larger than 4 cm (*p* = 0.002; mean size 5.6 cm vs. 3.028 cm, *p* = 0.011) (Table 2).

### 2.2. TERT mRNA Expression is More Frequently Found in Malignant Tumours, Being Associated with Worse Prognosis Features

To understand *TERT* re-expression in thyroid tumours, independently of its mechanism of reactivation, *TERT* mRNA expression was analysed. Benign and malignant tumours were positive for *TERT* mRNA expression, however the frequencies in which the expression was detected in each group were significantly different (*p* < 0.0001). *TERT* mRNA expression was detected in 14 of the 83 benign tumours analysed (17% of the FTAs), whereas 47 of the 98 malignant samples were *TERT* mRNA positive (48%).

*TERT* mRNA expression (Table 3) was detected in 17% of the FTAs, in 50% of the FTCs, in 45% of the PTCs (46% in CV-PTC, 45% in FV-PTC, 40% in aggressive variants of PTC and 40% in OV-PTCs), 100% of the PDTCs and in the distant metastasis with *TERT*p mutation. The thyroid tissue adjacent to benign and malignant tumours was *TERT* mRNA positive in 29% and 39% of the cases, respectively.

**Figure A1:**
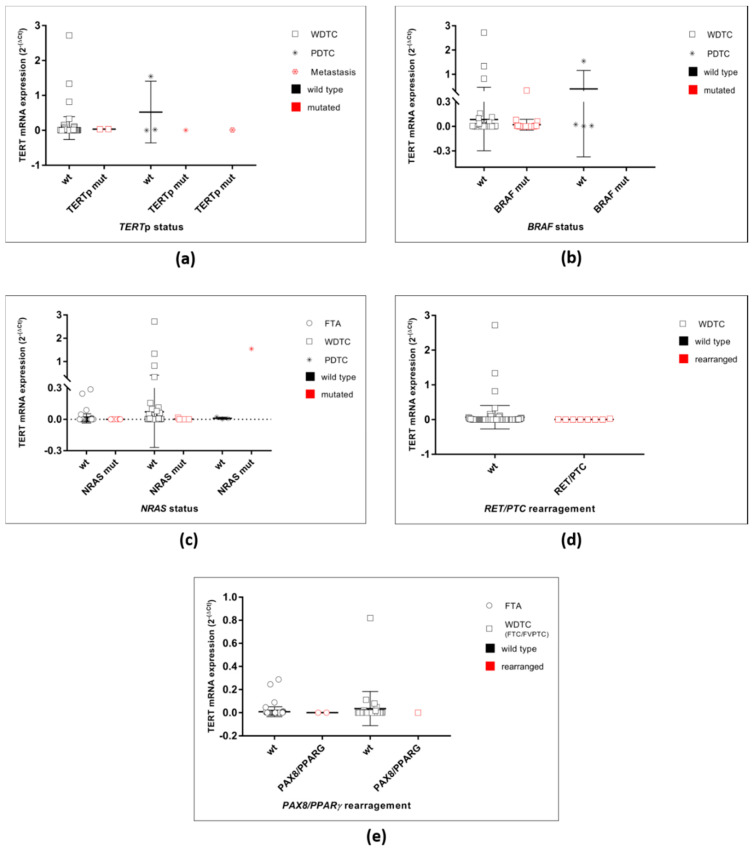
TERT mRNA expression in the malignant tumours grouped by genetic alterations. (**a**) TERT mRNA expression in all malignant cases grouped according to the TERTp status as wild-type or mutated. (**b**) TERT mRNA expression in the WDTC and PDTC grouped according to the BRAF status as wild-type or mutated. No significant differences were found. (**c**) TERT mRNA expression in the WDTC and PDTC grouped according to the NRAS status as wild-type or mutated. No significant differences were found. (**b**) TERT mRNA expression in the WDTC grouped according to the RET/PTC status. No significant differences were found. (**c**) TERT mRNA expression in the FTA and WDTC grouped according to the PAX8/PPARγ status. No significant differences were found.

All cases with *TERT*p mutation were positive for *TERT* mRNA expression (*p* = 0.049), with no significant differences regarding the levels of expression between the tumours with or without *TERT*p mutation (Figure A1). Malignant tumours from older patients present more frequently *TERT* expression (67% positive tumours vs. 33% negative tumours, *p* = 0.044) (Table 4). In the benign lesions *TERT* mRNA positive tumours were associated with the presence of lymphocytic infiltrate (*p* < 0.0001), 85% of the FTAs (11 cases) with positive *TERT* mRNA expression had lymphocytic infiltrate vs. 15% (2 cases) negative for the presence of lymphocytic infiltrate (Table 4).

**Figure 1:**
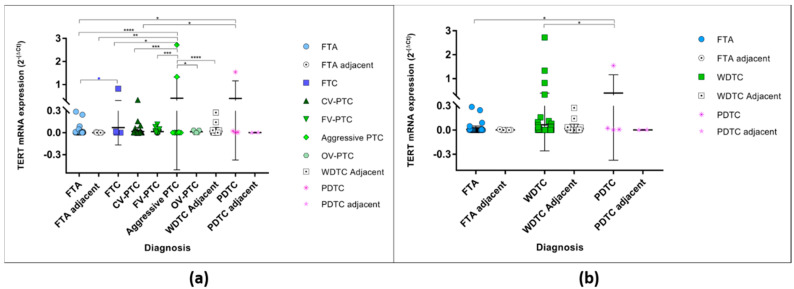
*TERT* mRNA levels of expression in all studied samples according to the histological diagnosis. (**a**) *TERT* mRNA expression in benign and malignant tumours and their respective adjacent thyroid. * One-way ANOVA (Bonferroni correction) statistical significance (*p* < 0.0001); * unpaired *t*-test statistical significance (*p* = 0.0294). (**b**) *TERT* mRNA expression grouped in major categories, the FTA and their respective adjacent thyroid, and the malignant, the WDTC (well differentiated follicular derived thyroid carcinomas—FTC, CV-PTC, FV-PTC, aggressive variants of PTC, and OV-PTC) and their respective adjacent thyroid, and the PDTCs and respective adjacent thyroid. * One-way ANOVA (Bonferroni correction) statistical significance (*p* = 0.0086). * *p* < 0.05, ** *p* < 0.01, *** *p* < 0.001, **** *p* < 0.0001.

When the expression levels were compared, the expression between diagnosis was significantly different (Figure 1a) (One-way ANOVA *p* < 0.0001), the aggressive variants of PTC and PDTCs being the histological groups with the highest levels of *TERT* mRNA expression. The aggressive variants of PTC (2 cases of tall cell PTC, 1 case diffuse sclerosing PTC and 1 case trabecular/solid PTC) had significant higher levels of expression than the FTAs, FTCs, CV-PTC, FV-PTC and OV-PTCs, whereas the PDTCs had higher levels of expression in comparison to FTAs and CV-PTC. Both the aggressive variants of PTC and PDTCs had higher levels of expression than the adjacent thyroid. For analysis purposes, the malignant WDTC were grouped in one major category, which included all the PTCs and its variants and the FTC tumours. When these groups (FTA, WDTC, and PDTCs) were compared the expression was significantly different (One-way ANOVA *p* = 0.0086). The PDTCs were the group with the highest level of expression, being significantly increased when compared with the FTA and WDTC (Figure 1b).

**Figure 2:**
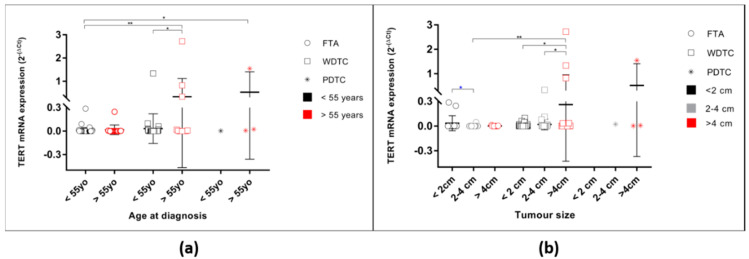
*TERT* mRNA levels of expression in all studied samples according to age at diagnosis and tumour size. (**a**) *TERT* mRNA expression of FTA, WDTC, and PDTC cases grouped by age at diagnosis. * One-way ANOVA (Bonferroni correction) statistical significance (*p* = 0.0005); (**b**) *TERT* mRNA expression of FTA, WDTC, and PDTC cases grouped by tumour size. * One-way ANOVA (Bonferroni correction) statistical significance for the FTA (*p* = 0.0440); * unpaired *t*-test statistical significance between FTA < 2cm and FTA 2–4cm (*p* = 0.0331). * One-way ANOVA (Bonferroni correction) statistical significance for WDTC (*p* = 0.0209). * One-way ANOVA (Bonferroni correction) statistical significance for the FTA and WDTC (*p* = 0.0058). * *p* < 0.05, ** *p* < 0.01.

Since *TERT* mRNA status was associated with age at diagnosis in the malignant tumours, the levels of expression were assessed considering this feature, for both benign and malignant tumours (One-way ANOVA *p* = 0.0005). In the FTAs (Figure 2a), there were no differences regarding the levels of expression between patients younger or older than 55 years old. In the malignant tumours, however, the highest levels of expression were found in tumours belonging to patients older than 55 years old, the difference being significant when considering the WDTC.

**Figure A2:**
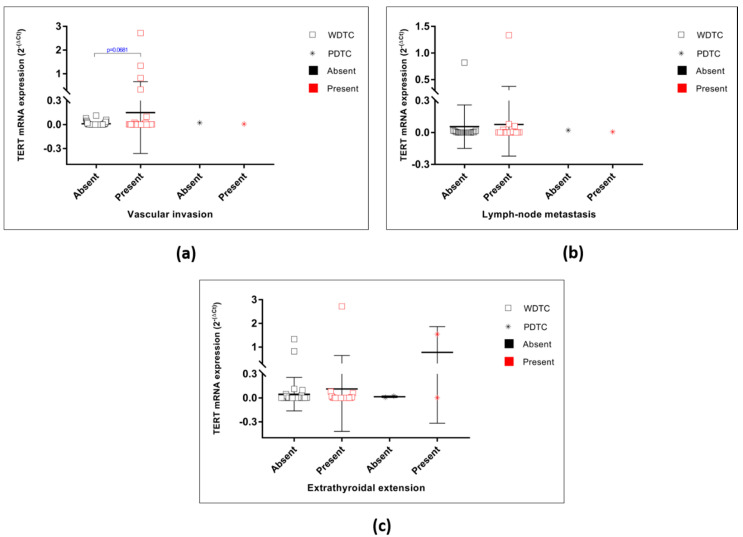
TERT mRNA expression in the malignant tumours considering several clinico-pathological features. (**a**) TERT mRNA expression in the WDTC and PDTC grouped by vascular invasion status (*p* = 0.0681). No significant differences were found. (**b**) TERT mRNA expression in the WDTC and PDTC grouped by lymph-node metastasis. No significant differences were found. (**c**) TERT mRNA expression in the WDTC and PDTC grouped by minimal miETE. No significant differences were found.

*TERT* mRNA levels of expression were also significantly different when considering the tumour size (Figure 2b, ANOVA *p* = 0.0008). In the benign tumours, the levels of expression were significantly different (One-way ANOVA *p* = 0.0440), the FTAs smaller than 2 cm being the ones with the highest levels of expression. In the malignant tumours, *TERT* mRNA level of expression in WDTC were also different when grouped by tumour size at diagnosis (One-way ANOVA *p* = 0.0209). At variance with the benign tumours, in malignant tumours the highest levels of expression were detected in the larger tumours. Additionally, there was a tendency to higher *TERT* mRNA expression in the WDTC presenting vascular invasion (*p* = 0.0661; Figure A2). Furthermore, when considering the tumours with follicular pattern (FTC and encapsulated FV-PTC, *n* = 21), there was a significant difference, being the levels of *TERT* mRNA increased in the tumours that presented vascular invasion (*p* = 0.0446).

**Figure 3:**
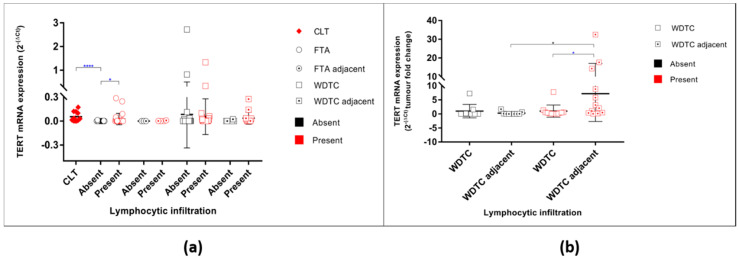
TERT mRNA levels of expression in all studied samples according to the presence of lymphocytic infiltration. (**a**) TERT mRNA expression in benign and malignant tumours and their respective adjacent thyroid counterparts regarding the absence/presence of lymphocytic infiltration. * represents the statistical significance according to *t*-test for independent samples; (**b**) Adjacent thyroid fold change relative to its respective primary tumour grouped by lymphocytic infiltration status. * represents the statistical significance according to One-way ANOVA (Bonferroni correction) test (*p* = 0.0173); * represents the statistical significance according to *t*-test for independent samples (*p* = 0.0457). * *p* < 0.05, **** *p* < 0.0001.

The FTAs that present lymphocytic infiltration had significantly higher levels of *TERT* mRNA expression (unpaired *t*-test *p* = 0.0214) (Figure 3a) than FTA without that feature. To understand the association of higher levels of *TERT* mRNA in the FTAs and WDTC with lymphocytic infiltrate, 11 cases of CLTs (without associated lesions) were analysed. All the CLT cases were positive for *TERT* mRNA expression, the levels being significantly higher than those in the FTAs without lymphocytic infiltration (unpaired *t*-test *p* < 0.0001) and similar to the FTAs with lymphocytic infiltration (Figure 3a).

There were no significant differences in *TERT* mRNA levels in the malignant tumours when categorized by the absence or presence of lymphocytic infiltration (Figure 3a). No significant differences were found between the WDTC and respective adjacent thyroid regarding the levels of *TERT* mRNA expression in the tumours without lymphocytic infiltration (Figure 3). However, in the WDTC with lymphocytic infiltration, the adjacent thyroid had a significant increase expression of *TERT* mRNA (unpaired *t*-test *p* = 0.0457) when compared with the respective primary tumour and when compared with the adjacent thyroid of malignant tumours without lymphocytic infiltration; (One-way ANOVA *p* = 0.0173; Figure 3b).

## 3. Discussion

The presence of *TERT*p mutations in thyroid cancer has been associated with worse prognostic features, such as older age, larger tumour size, higher tumour stage, as well as with the presence of distant metastasis, worse response to treatment, and short survival [19,20,23,24,25,26,33,34]. In this work we aimed to evaluate both *TERT*p mutations and *TERT* mRNA features in a series of benign and malignant thyroid lesions, representing a series without aggressive features. The presence of *TERT*p mutations was an uncommon finding in our series, whereas the expression of *TERT* mRNA was a common event in the malignant tumours (48% of the cases) and present in the benign tumours (17%).

The presence of *TERT*p mutations only in primary tumours with more aggressive features (PDTC or larger tumours) and in one lymph node metastasis and one distant metastasis, confirms the low frequency of these mutations in series of non-aggressive tumours, and in an indirect way confirm that these mutations are closely associated with worse prognosis features, as previously reported by our group [24,33,34]. Furthermore, considering all the mutated samples we also verified the association of *TERT*p mutations with older age at diagnosis and larger size of tumours, both features of poor prognosis in thyroid cancer.

Contrary to other reports in the literature that described *TERT*p mutations in benign lesions [27,28,29], no *TERT*p mutations were found in our large series of 87 sporadic FTAs. In a sporadic context, one study reported the presence of *TERT*p mutations in 2% of the FTA and 17% of atypical FTA and another study reported one case of FTA with *TERT*, *HRAS*, and *EIF1AX* mutations [28] which lead the authors to conclude that this could be an early event in thyroid tumours that still had not developed malignant features [29]. In line with the previous results from our group [24,35], and in accordance with previous studies [30,36], in the present work we did not find evidence to support these conclusions.

In the current work the presence of *BRAF* mutations, whether considering only the PTCs or the whole series, was significantly associated with miETE confirming the association of these genetic alterations with local invasiveness. *BRAF* has been addressed as a prognostic marker in thyroid cancer in the literature, where associations were found with larger tumours, older age, miETE, and lymph node metastasis [37,38,39,40]. Nonetheless, several other works have refuted these associations [34,41,42] and in the present work we did not confirm the association of *BRAF* with worse prognostic features.

Remarkably, the FCT tumour positive for PAX8/PPARγ rearrangement also harboured a RET/PTC1 rearrangement. It has been described that the RET/PTC rearrangement distribution may be quite heterogenous, being detected in almost all neoplastic cells, or being in only a few tumour cells, the so called non-clonal *RET/PTC* [43]. Our hypothesis is that this co-occurrence of both rearrangements can reflect the morphologic differentiation of the tumour, with some areas of the tumours having a solid architecture, positive for the RET/PTC rearrangement, and other areas with follicular architecture, positive for *PAX8/PPAR*γ, which would enable the identification of the rearrangements in the same tumour [44].

Even in the absence of the *TERT*p mutations, benign and malignant thyroid tumours had *TERT* mRNA expression. *TERT* mRNA expression was more frequently detected in the malignant tumours than in the benign tumours, as previously described in other works [14,15,16,17,18]. All the *TERT*p mutated cases were positive for *TERT* mRNA expression; however, these cases did not show higher levels of expression of *TERT* mRNA when compared with tumour wild type for *TERT*p mutations. Even though the series is small, it is possible to report that malignant thyroid tumours are frequently *TERT* mRNA positive in the absence of hotspot *TERT*p mutations and the mechanism by which *TERT* re-expression occurs in these cases remains largely unknown. A recent study reported that in thyroid cancer, the hotspot mutations c.1−124C > T and c.1−146C > T are the most frequently detected mutations, but there are other alterations in the promoter [45]. Supporting the latter point, one study analysed several *TERT* genetic alterations, including the *TERT*p mutations, *TERT* mRNA expression, *TERT*p hypermethylation and *TERT* gene copy number in follicular patterned tumours (FTC, FTA and atypical FTA). The authors reported other *TERT* aberrancies than the *TERT*p mutations [30]. Therefore, there are other TERT genetic alterations, e.g., other promoter mutations, methylation of regions of the promoter, gene amplification or rearrangements that can lead to *TERT* transcriptional activation, that need to be further explored. Of note, *TERT* mRNA expression levels were not different when considering other genetic alterations present in the tumours, such as *BRAF*, *NRAS* mutations and *RET/PTC* or *PAX8/PPARγ* rearrangements (Figure A1).

*TERT* mRNA expression was more frequently detected in the more aggressive tumours, being the highest levels of expression found in the aggressive variants of PTC and in PDTCs. Similar to the *TERT*p mutations [24], *TERT* mRNA expression was associated with older age at diagnosis and larger size of tumours and there was a tendency towards association between higher levels of *TERT* mRNA expression and the presence of vascular invasion. It seems that *TERT* mRNA expression is also associated with worse prognosis features in thyroid cancer, similar to the presence of *TERT*p mutations, as advanced in previous reports [16,46,47,48,49].

Nevertheless, our results show that expression of *TERT* mRNA is not exclusive for malignant thyroid tumours, in accordance with previous studies reporting *TERT* mRNA in FTAs, hyperplasia, goiter and Graves’ disease [14,15,16]. Contrary to the malignant tumours, there were no associations with age at diagnosis. Regarding tumour size, it was possible to establish opposite behaviours in the benign and malignant tumours, the benign tumours had the highest levels of *TERT* expression in the smaller tumours, whereas the malignant had the highest levels of expression in the larger tumours. 

In our series 85% of the FTAs positive for *TERT* mRNA expression presented concomitant lymphocytic thyroiditis, which was in accordance with the Saji M et al. study, in which all the FTAs that had *TERT* expression had concomitant lymphocytic thyroiditis [16]. Lymphocytes are one of the cell lineages that maintain *TERT* expression after terminal differentiation [50]. Therefore, we hypothesized that *TERT* mRNA in the majority of these benign tumours, resulted from the presence of lymphocyte infiltration of the tumour sample. This assumption can also explain why the highest levels of *TERT* expression were found in the smaller tumours; this could represent a diminished ratio of tumour cells/lymphocytes, enhancing the levels of detection of *TERT* expression.

In order to test this hypothesis, frozen tissues from CLT were analysed and all of them were positive for *TERT* mRNA expression. We found also that FTA with lymphocytic infiltrate presented significant higher levels of *TERT* mRNA than FTA without this feature confirming that lymphocytic infiltrate may be responsible for the *TERT* mRNA positive expression in the FTAs setting. The presence of lymphocytic infiltration not only affects the evaluation of *TERT* mRNA expression in benign tumours. Thyroid samples adjacent to malignant tumours that present lymphocytic infiltration had higher levels of *TERT* mRNA expression than the tumour by itself and then thyroid adjacent to malignant tumours without lymphocytic infiltration. In thyroid fine-needle aspiration cytology materials the authors reported that 35% of the benign lesions were *TERT* positive, *TERT* sensitivity being increased in management of suspicious thyroid tumours when concomitant lymphocytic thyroiditis were excluded [51]. Detection of TERT expression in the tissue, by immunohistochemistry (if reliable antibodies are available) and/or fluorescence in situ hybridization [52], as a complementary analysis would allow the pathologist to distinguish the expression in tumour cells from the lymphocytic infiltration, and increase the possible use of *TERT* expression in the prediction/diagnosis of thyroid disease.

Only two cases diagnosed as FTA, were positive for *TERT* mRNA expression in the absence of lymphocytic infiltration. One belonged to a female, 50 years old, with a 4.5 cm tumour and with a smaller FTA in the adjacent thyroid parenchyma. The total inclusion of the capsule excluded capsular invasion. Considering the age of the patient and size of the tumour, we may hypothesize that this was in fact a borderline follicular patterned tumour. The second case belonged to a 34 years old male patient, with a 5 cm tumour, which was suggested, upon diagnosis revision [53], as a Non-invasive Follicular Thyroid Neoplasm with Papillary-Like Nuclear Features (NIFTP). *TERT* mRNA expression without the concomitance of lymphocytic infiltration, may indicate tumours be followed with caution. These results are in accordance with previous studies, that reported in FT-UMPs that the presence of *TERT*p mutations or other *TERT* aberrancies could identify a subset of tumours with higher malignant potential [30,31].

Summing up, *TERT*p mutations were uncommon findings in sporadic non-aggressive thyroid tumour series, occurring in aggressive malignant tumours. Still, even in a low frequency, the presence of *TERT*p mutations were associated with worse prognosis features, such as older age at diagnosis and larger size of tumours.

*TERT* mRNA expression is present both in benign and malignant tumours, with a higher frequency in the malignant tumours, even in the absence of *TERT*p mutations. *TERT* expression in follicular cell derived thyroid carcinomas has similar associations as the *TERT*p mutations, the highest levels of expression being found in aggressive histotypes, larger size of tumours, and from older patients. On the other hand, the presence of *TERT* expression in benign tumours can be due to the presence of lymphocytic infiltrate and lead to dubious interpretations. 

## 4. Materials and Methods 

### 4.1. Samples

The frozen samples were obtained from the Centro Hospitalar de São João (CHSJ) and kept at a temperature of −80 °C at the Institute of Molecular Pathology and Immunology of the University of Porto (Ipatimup). All clinico-pathological data were obtained from the anatomic pathology reports provided by the Department of Pathology from the CHSJ. The cases with available paraffin embedded blocks were revised and histological diagnosis were reported according to the strict histomorphological criteria for current World Health Organization terminology [13].

From the anatomic pathology reports the following clinico-pathological parameters were collected: diagnosis, age at diagnosis, gender, tumour size, presence of tumour capsule, presence of capsular invasion, associated lesions, vascular invasion, lymph node metastasis, extrathyroidal invasion, presence of lymphocytic infiltrate, and other histological observations.

The repository consisted of 500 cases which included hyperplastic, benign, and malignant thyroid lesions. According to the availability of enough frozen tissue, pathological report and/or clinical information, 244 samples from 202 patients were selected for the subsequent study. Those samples correspond to 11 samples of CLT; 87 samples of FTA (adjacent tissue available in 7 cases); 13 cases of FTC (adjacent tissue available in 8 cases); 85 cases of PTC (adjacent tissue available in 23 cases); 5 cases of PDTC (adjacent tissue available in 2); 2 lymph node metastasis and 1 distant metastasis.

Our study was conducted in accordance with the Declaration of Helsinki, and the protocol was approved in 2013 by the Ethical Committee of the Centro Hospitalar de São João (CHSJ, approval number CES284-13). Since it was an anonymized retrospective study it was exempted from the informed consent from each patient, according to national ethical guidelines.

### 4.2. RNA Extraction and cDNA Preparation

The samples were thawed at room temperature and fragments with about 1 cm were taken from each sample. Immediately after the selection the fragments these were homogenized in 2 mL of TRIzol^®^ Reagent (Life Technologies™, GIBCO BRL, Carlsbad, CA) and stored at −80 °C until further processing (DNA/RNA extraction.).

The RNA extraction was performed according to the manufacturer protocol [54], with the purpose of extracting from 1 mL of the homogenized tissue RNA and DNA from the same fragment. With this aim a chloroform phase separation was performed by the addition of chloroform to the sample and mixed. Samples were centrifuged and the aqueous phase was removed to a new tube containing isopropanol for RNA extraction and the remaining two phases (interphase and organic) were stored for DNA extraction.

The remaining RNA extraction steps and washes were performed according to the manufacturer’s protocol [54]. The RNA pellet was dissolved in DNase and RNase free water, quantified by Nanodrop N-1000 Spectrophotometer (Thermo Scientific, Wilmington, DE, USA) and stored at −80 °C. For cDNA preparation, 1 μg of total RNA was treated with DNase and reverse transcribed using the RevertAid first strand cDNA synthesis kit (Thermo Scientific/Fermentas, Waltham, MA, EUA), according to the manufacturer’s protocol. Of the synthetized cDNA an RT-PCR was performed for a house-keeping gene, the β-actin gene, to access the quality of the sample, confirmed by positive amplification at CT between 15–25.

### 4.3. DNA Extraction

DNA extraction was performed with the two remaining phases, that were again centrifuged (12,000× *g* for 15 minutes at 4 °C). Analytical grade absolute ethanol was used to precipitate DNA, and the sample was gently homogenized by inversion followed by an incubation of 2–3 min at room temperature. The sample was then centrifuged (12,000× *g* for 10 min at 4 °C), the supernatant discarded, and the pellet dried for a few seconds in a hot plate at 55 °C. Lysis Solution (Citogene^®^, Citomed, Lisbon, Portugal, EU) was added and the pellet homogenized by vortex. The sample was incubated overnight at 55 °C with shaking after Proteinase K (20 mg/mL) was added. When total digestion was achieved, the samples was cooled to RT and Protein Precipitation Solution (ref PP-125, Citogene^®^, Citomed, Lisbon, Portugal, EU) was added and incubated in ice for 10 min. Samples were centrifuged (16,000× *g* for 3 min at 0 °C) and the supernatant was transferred to a new tube containing analytical grade absolute isopropanol and glycogen (Thermo Scientific, Waltham, MA, EUA) and homogenized by gentle inversion at least 50 times. This step was followed by centrifugation (16,000× *g* for 3 min at 15 °C), and the supernatant was discarded. The DNA pellet was washed with Analytical grade 70% ethanol with homogenization of the pellet by gentle inversion. The sample was again centrifuged (16,000× *g* for 3 min at 15 °C) and supernatant discarded. The pellet was dried on a hot plate at 55 °C for 10 s and dissolved in DNase and RNase free water. DNA quantification was performed in Nanodrop N-1000 Spectrophotometer (Thermo Scientific, Wilmington, DE, USA) and stored at −20 °C.

### 4.4. Genetic Alterations: PCR and Sanger Sequencing

The genetic screening for hotspot mutations in *BRAF* (exon 15), *NRAS* (exon 2) and *TERT* (promoter region) was performed by Polymerase Chain Reaction (PCR) using GoTaq^®^ G2 Flexi DNA Polymerase (Promega, Madison, WI, USA), as previously described in Castro et al. [55] and Vinagre, et al. [35].

Synthetized cDNA was analysed by PCR using GoTaq^®^ G2 Flexi DNA Polymerase (Promega, Madison, WI, USA) for the presence of PAX8-PPARɣ and RET/PTC rearrangements according to the procedures described by Marques, et al. [56] for *PAX8-PPARɣ* and Lima, et al. [57] for *RET/PTC1* and *RET/PTC3*. 

### 4.5. Quantitative PCR Analysis

*TERT* mRNA expression was analysed by qPCR using TaqMan PCR Master Mix (Applied Biosystems, Foster City, CA, USA) and the amplification level was detected by an ABI PRISM 7500 Fast Sequence Detection System (Applied Biosystems), that was programmed to an initial step of 2 min at 50 °C, 10 min at 95 °C, followed by 45 cycles of 95 °C for 15 s and 60 °C for 1 min. Probes used for this analysis were: *TERT* (IDT Coralville, IA, USA; no. HS.PT.56a.40988589) and as endogenous control *huTBP* gene (TATA-binding protein) (IDT Coralville, IA, USA; no. Hs.PT.39a.22214825).

Relative quantification of target genes was determined using the ΔΔCT method, where similar amplifications efficiencies between *TERT* mRNA and *huTBP* were obtained, by Livak’s Linear Regression Method (slope = 0.0696) (Sequence Detector User Bulletin 2; Applied Biosystems).

### 4.6. Statistical Analysis

Statistical analysis was performed using IBM SPSS Statistics version 25 (IBM, Armonk, NY, USA), where Chi-Square, with Fishers correction, unpaired *t*-test and One-way ANOVA (post-hoc Bonferroni) were used to compare the groups. This program was used considering the analysis of the relationship between patient’s gender, age, tumour size, diagnosis, histological characteristics, molecular status, and TERT mRNA expression status, with the use of unpaired *t*-test, Mann–Whitney test and Chi-Square with Fisher’s correction.

GraphPad Prism version 7.0 (GraphPad Software, Prism, La Jolla California, CA, USA) using unpaired *t*-test and One-way ANOVA (post-hoc Bonferroni) were used to evaluate *TERT* mRNA levels of expression considering the different of clinico-pathological features and to build the graphic representation of the results.

## 5. Conclusions

In conclusion, we report that *TERT* expression may be correlated with worse prognosis features in malignant thyroid tumours but its expression in benign thyroid tumours should be carefully considered and analysed, particularly in the presence of lymphocytic infiltrate.

## Figures and Tables

**Table 1:** Clinico-Pathological and Genetic Characterization of the Series, with Total Number of Samples for Each Diagnosis and Frequencies of the Presence of Invasion or Genetic Alterations.

	Variables	Benign		Malignant					
		**FTA**	**CLT**	**FTC**	**CV-PTC**	**FV-PTC**	**Aggressive Variants of PTC**	**OV-PTC**	**PDTC**
Clinical-pathological variables	N	87	11	13	48	21	11	5	5
	Adjacent thyroid tissue	7	-	8	12	7	3	1	2
	Age (mean)	42.8	51.3	46.1	38.4	42.4	45.8	50.8	65.6
	Gender F *n* (%)	70 (82.4)	11 (100.0)	10 (76.9)	38 (82.6)	19 (90.5)	7 (63.6)	5 (100.0)	3 (60.0)
	Tumour size (mean)	3.3	-	3.6	2.4	3.3	3.8	2.7	5.1
	Lymphocytic infiltrate *n* (%)	29 (37.7)	-	3 (23.1)	21 (51.2)	8 (42.1)	5 (45.5)	3 (75.0)	0 (0.0)
	Vascular invasion *n* (%)	-	-	4 (30.8)	23 (56.1)	5 (26.3)	6 (60.0)	0 (0.0)	2 (66.7)
	Lymph node metastasis *n* (%)	-	-	0 (0.0)	15 (68.2)	3 (60.0)	4 (80.0)	0 (0.0)	2 (66.7)
	Minimal extrathyroidal extension *n* (%)	-	-	0 (0.0)	17 (42.5)	5 (26.3)	7 (63.6)	0 (0.0)	2 (40.0)
Genetical characterization	*TERT*p *n* (%)	0 (0.0)	-	0 (0.0)	0 (0.0)	0 (0.0)	0 (0.0)	2 (40.0)	1 (20.0)
	*BRAFn* (%)	0 (0.0)	-	0 (0.0)	20 (41.7)	3 (14.3)	3 (27.3)	0 (0.0)	0 (0.0)
	*NRASn* (%)	6 (6.9)	-	2 (15.4)	3 (6.3)	2 (9.5)	0 (0.0)	2 (40.0)	1 (20.0)
	*RET/PTCn* (%)	0 (0.0)	-	1 (8.3)	6 (12.5)	0 (0.0)	2 (20.0)	0 (0.0)	0 (0.0)
	*PAX8/PPARγn* (%)	2 (2.4)	-	1 (8.3)	0 (0.0)	0 (0.0)	0 (0.0)	0 (0.0)	0 (0.0)

**Legend:** FTA—Follicular thyroid adenoma; CLT—Chronic lymphocytic thyroiditis; FTC—Follicular thyroid carcinoma; CV-PTC—classic variant of papillary thyroid carcinoma; FV-PTC—Follicular variant of papillary thyroid carcinoma; Aggressive variants PTC—Aggressive variants of papillary thyroid carcinoma, that comprise tall cell, diffuse sclerosing, and trabecular variants; OV-PTC—oncocytic variant of papillary thyroid carcinoma; PDTC—Poorly differentiated thyroid carcinoma; TERTp—*TERT* promoter hotspots point mutations; BRAF—hotspots in exon 15 screening (*BRAF* V600E); NRAS—hotspots *NRAS* exon 2 screening (codon 61); RET/PTC—presence of the *RET/PTC* rearrangements; PAX8/PPARγ—presence of the *PAX8/PPARγ* rearrangement.

**Table 2:** Association of *TERT*p mutations with mean age at diagnosis and mean tumour size considering all the available malignant samples (*n* = 103).

Clinico-Pathological Features		*TERT*p Mutations		
		wt	Mutated	*p*-Value
Age	<55 years *n* (%)	75 (78.1)	1 (25.0)	0.042
	>55 years *n* (%)	21 (21.9)	3 (75.0)	
	Mean age	42.50	60.00	0.039
Tumour size	<2 cm *n* (%)	35 (39.3)	0 (0.0)	0.002
	2–4 cm *n* (%)	34 (38.2)	0 (0.0)	
	>4 cm *n* (%)	20 (22.5)	4 (100.0)	
	Mean size	3.028	5.575	0.011

**Table 3:** Frequency of Samples Positive for TERT mRNA Expression Grouped by Diagnosis.

Diagnosis	TERT mRNA Expression *n* (%)
CLT	11/11 (100.0)
FTA	14/83 (16.9)
FTA adjacent tissue	2 */7 (28.6)
FTC	6/12 (50.0)
CV-PTC	21/45 (45.7)
FV-PTC	9/20 (45.0)
Aggressive variants of PTC	4/10 (40.0)
OV-PTC	2/5 (40.0)
PDTC	4/4 (100.0)
Thyroid tissue adjacent to malignant tumours	13/33 (39.4)

Legend: CLT—Chronic lymphocytic thyroiditis; FTA—Follicular thyroid adenoma; FTC—Follicular thyroid carcinoma; CV-PTC—classic variant of papillary thyroid carcinoma; FV-PTC—Follicular variant of papillary thyroid carcinoma; Aggressive variants PTC—Aggressive variants of papillary thyroid carcinoma, that comprise tall cell, diffuse sclerosing, and trabecular variants; OV-PTC—oncocytic variant of papillary thyroid carcinoma; PDTC—Poorly differentiated thyroid carcinoma. * Two adjacent thyroids were paired to FTAs with lymphocytic infiltration (both tumour and adjacent tissue were *TERT* mRNA positive). Note—Four benign samples and seven malignant samples were excluded due to lack of RNA integrity.

**Table 4:** Association of TERT mRNA Expression with Mean Age at Diagnosis and Lymphocytic Infiltration Status Considering all the FTA and Malignant Samples.

Clinico-Pathological Features		FTA			Malignant Tumours		
		*TERT* mRNA Expression			*TERT* mRNA Expression		
		Negative	Positive	*p*-Value	Negative	Positive	*p*-Value
Age at diagnosis	<55 years *n* (%)	55 (85.9)	9 (14.1)	0.109	40 (57.1)	30 (42.9)	0.044
	>55 years *n* (%)	11 (68.8)	5 (31.3)		8 (33.3)	16 (66.7)	
	Age (mean)	41.6	48.3	0.09	38.90	48.1	0.008
Lymphocytic infiltration	Absent *n* (%)	45 (95.7)	2 (4.3)	0.000	28 (58.3)	20 (41.7)	0.311
	Present *n* (%)	16 (59.3)	11 (40.7)		18 (47.4)	20 (52.6)
